# Association of Pancreatic Fatty Infiltration With Age and Metabolic Syndrome Is Sex-Dependent

**DOI:** 10.1016/j.gastha.2022.01.007

**Published:** 2022-03-30

**Authors:** Sameer Bhalla, George A. Kuchel, Stephen Pandol, Faraz Bishehsari

**Affiliations:** 1 Division of Digestive Diseases and Nutrition, Department of Internal Medicine, Rush University Medical Center, Chicago, Illinois; 2 UConn Center on Aging, University of Connecticut, Farmington, Connecticut; 3 Division of Digestive and Liver Disease, Cedars-Sinai Medical Center, Los Angeles, California; 4 Rush Center for Integrated Microbiome and Chronobiology Research, Rush University Medical Center, Chicago, Illinois

**Keywords:** Pancreatic Fat, Pancreatic Cancer, Metabolic Syndrome, Metabolic Dysfunction

## Abstract

**Background and Aims:**

Fatty infiltration of the pancreas has been shown to be associated with both precancerous pancreatic lesions and pancreatic ductal adenocarcinoma. We aim to determine predictors of fatty infiltration of the pancreas in United States adults.

**Methods:**

In this retrospective cohort study conducted at a large academic hospital in Chicago, Illinois, we calculated the relative fatty infiltration of the pancreas (corrected to spleen) of 265 cancer-free individuals based on their cross-sectional imaging. Demographic data and relevant laboratory results were obtained from medical records.

**Results:**

We found that age was the strongest predictor of fatty infiltration of the pancreas in our series (*P* < .01). Fatty infiltration of the pancreas was also significantly associated with body mass index (*P* < .01) and hyperlipidemia (*P* < .05). In women, in addition to age (*P* < .05), elevated body mass index (*P* = .023), hyperlipidemia (*P* = .013), and fatty liver (*P* = .017) were predictors of fat in pancreas. We found a sex-dependent association between pancreatic fat and metabolic syndrome including fatty liver (*P* = .002).

**Conclusion:**

Fatty infiltration of the pancreas increases by age and components of metabolic syndrome. These assertions could be sex-dependent.

## Introduction

Age is a well-established risk factor for pancreatic ductal adenocarcinoma (PDAC), a disease with a rising incidence and a poor prognosis.^1^ PDAC has a 5-year survival rate of only 9%^2^ largely due to its late diagnosis in advanced stages and resistance to chemotherapy, radiotherapy, and immunotherapy. Because the overall prevalence of PDAC is low and it is difficult to identify precancerous pancreatic lesions with available imaging modalities, it is imperative to identify specific risk factors that predispose an individual to develop PDAC. Recently, fatty infiltration of the pancreas (FIP) has gained significant interest in the pathophysiology of PDAC. FIP has been shown to be associated with both precancerous lesions (eg, pancreatic intraepithelial neoplasia)^3^ as well as PDAC^4^ in humans. While histopathological assessment of the tissue remains the gold standard of detecting fat in the pancreas, changes of pancreatic steatosis are well correlated with the attenuation of the pancreas on imaging studies such as computed tomography (CT), which can be used as a noninvasive method to estimate fat in the pancreas.^5^ In a recent case-control study, we found that the degree of FIP, as estimated by imaging, is an independent risk factor for PDAC.^6^ The predistortion of FIP to pancreatic cancer formation and progression is further supported by animal models.^7^ It is therefore important to identify factors that are associated with FIP.

Similar to other organs,^8^ FIP may increase by ageing, with ageing driving the process of fatty infiltration and consequently fatty infiltration, especially if accompanied by presence of senescent cells, further accelerating other ageing processes. This is supported by recent histological and imaging studies that showed that fatty tissue and ultrasound echogenicity of the pancreas, respectively, increased with age.^9^^,^^10^ Besides the association of FIP with ageing, FIP may correlate with metabolic risk factors and has been proposed to represent a manifestation of the metabolic syndrome. Metabolic syndrome, characterized by obesity, hypertension, and varied systemic metabolic dysfunctions, is associated with several comorbidities including fatty infiltration of liver.^11^ Nonalcoholic fatty liver disease, an increasingly common condition in industrial societies including the United States (U.S.), has been shown to be associated with increased risk of liver cancer^12^ and is estimated to be the major contributing factor to the burden of liver cancer by 2030.^13^ With regard to pancreatic cancer, and its increasing rates, accumulating evidence suggests that the rise of metabolic syndrome in recent generations could be a contributing factor to the uptrend observed in the incidence of PDAC in the U.S.^10^^,^^14^ Mechanisms via which metabolic syndrome facilitates pancreatic cancer progression and whether such mechanisms involve FIP still remain unclear.

FIP, similar to metabolic syndrome and fatty liver, is thought to be a common phenomenon, with estimated prevalence of ∼33% at the population level.^15^ Individuals with FIP are commonly found to have parameters of the metabolic syndrome,^16^, ^17^, ^18^ and a recent prospective study found that it is independently associated with subsequent diabetes mellitus (DM) development.^19^ Therefore, FIP could be a credible mechanism by which metabolic syndrome impinges on carcinogenesis in the pancreas. Risk of metabolic syndrome also increases with age.^20^ It is unclear to what extent FIP is due to ageing or secondary to metabolic syndrome associated with ageing. Here, using objective imaging criteria, we aimed to identify predictors of FIP in cancer-free subjects. We found that age was the strongest predictor of FIP in our series. The association of metabolic syndrome with FIP was age- and sex-dependent. These results have clinical implications for identifying risk factors of FIP.

## Methods

### Patient Selection

We expanded on the previously studied individuals with no history of malignancy who underwent CT colonography (also a noncontrast study), for colon cancer screening at the Rush University Medical Center over a 10-year period (2006–2016). Exclusion criteria included individuals with increased risk of pancreatic cancer, known pancreatobiliary pathology, and type 1 DM. Demographic data and relevant laboratory results (eg, age, sex, body mass index [BMI], type 2 DM, and liver function tests) were obtained from medical records. A method for measuring FIP has been previously described.^6^ Briefly, we followed the radiological method of quantifying pancreatic fat from the study by Kim et al,^21^ which was corroborated by comparing CT and histological measurements of pancreatic fat. We measured the area (1.0 cm^2^) of pancreatic attenuation (P) in 3 regions including the pancreatic head, body, and tail to calculate a mean attenuation. The splenic attenuation (S) was similarly measured, and P was corrected to the individual’s S without the use of intravenous contrast. On CT, areas of FIP appear hypoattenuated relative to normal tissue. We calculated P minus S and added 100 to each pure P minus S value to ensure positive values—labeled as PS100. The PS100 value was used as an estimate FIP, with lower PS100 translating to a higher relative FIP.

### Statistical Analysis

Statistical Package for the Social Sciences v23 (IBM SPSS Statistics, Chicago, IL) and R Studio v3.5.1 were used for all analyses. Univariate analyses were performed to determine independent risk factors for FIP. Pearson correlation was performed to determine the interaction between PS100 and metabolic syndrome variables in men and women older and younger than 70 years. Analysis of variance and student’s t-test were performed to determine differences in PS100 among different age groups and those with/without metabolic risk factors. Given that fatty liver may be considered an additional feature of metabolic syndrome and related to FIP, we defined metabolic syndrome including fatty liver (MetL) as presence of 3 or more of the following factors, BMI > 25, hypertension, low high-density lipoprotein cholesterol (<40 mg/dL in men, <50 mg/dL in women), triglycerides ≥ 150 mg/dL, DM, and fatty liver,^22^, ^23^, ^24^ and examined the association of FIP with MetL in each sex, after adjusting for age.

## Results

The demographic data of 265 individuals with available imaging in our system are separated by age (above and below 70 years) and sex and displayed in Table 1. The association of patient’s metabolic factors with pancreatic fat is shown in Table 2. Age (*P* < .01) was the strongest independent predictor of PS100. Patients with any component of metabolic syndrome showed an overall lower PS100 than patients without that component of metabolic syndrome, with PS100 being significantly associated with BMI (*P* < .01) and hyperlipidemia (*P* < .05). We have observed a significant interaction between sex and having a component of metabolic syndrome (versus those without) (*P* = .02). We then analyzed our data in men and women separately (Table 3), which showed that in women, in addition to age (*P* < .05), elevated BMI (*P* = .023), hyperlipidemia (*P* = .013), and fatty liver (*P* = .017) (an established complication of metabolic syndrome) were predictors of FIP. Women who had 3 or more components of metabolic syndrome tended to have higher FIP (*P* = .06). Three or more components of MetL were associated with higher FIP in women, and not in men.

**Table 1:** Demographic Data Separated by Age (Above and Below 70) and Sex (N = 265)

Demographic	All	Male age < 70 y	Male age ≥ 70 y	Female age < 70 y	Female age ≥ 70 y	*P*
n (%)	255 (100)	39 (15.3)	28 (11.0)	120 (47.1)	68 (26.7)	
Age (SD)	65.85 (12.23)	58.13 (9.31)	77.96 (6.25)	58.79 (8.55)	77.75 (6.60)	<.001
Body mass index (SD)	29.10 (7.80)	30.06 (5.24)	26.98 (4.85)	30.11 (9.55)	27.46 (5.64)	.10
Ethnicity						.30
White, n (%)	122 (47.8)	20 (51.3)	15 (53.6)	52 (43.3)	35 (51.5)	
African American, n (%)	87 (34.1)	9 (23.1)	9 (32.1)	51 (42.5)	18 (26.5)	
Hispanic, n (%)	5 (2.0)	2 (5.1)	0 (0)	3 (2.5)	0 (0)	
Other, n (%)	6 (2.4)	1 (2.6)	1 (3.6)	2 (1.7)	2 (2.9)	
Unknown, n (%)	35 (13.7)	7 (17.9)	3 (10.7)	12 (10.0)	13 (19.1)	
Tobacco use, n (%)						.23
Current	24 (9.4)	3 (7.7)	1 (3.6)	15 (12.5)	5 (7.4)	
Former	85 (33.3)	15 (38.5)	10 (35.7)	34 (28.3)	26 (38.2)	
Never	105 (41.2)	14 (35.9)	13 (46.4)	57 (47.5)	21 (30.9)	
Unknown	41 (16.1)	7 (17.9)	4 (14.3)	14 (11.7)	16 (23.5)	
Alcohol abuse, n (%)						.37
Current	4 (1.6)	0 (0)	0 (0)	3 (2.5)	1 (1.5)	
Former	9 (3.5)	2 (5.1)	2 (7.1)	5 (4.2)	0 (0)	
Never	201 (78.8)	30 (76.9)	22 (78.6)	98 (81.7)	51 (75.0)	
Unknown	41 (16.1)	7 (17.9)	4 (14.3)	14 (11.7)	16 (23.5)	
Family history of gastrointestinal cancer, n (%)						.67
Yes	55 (21.6)	6 (15.4)	6 (21.4)	27 (22.5)	16 (23.5)	
No	156 (61.2)	26 (66.7)	16 (57.1)	77 (64.2)	37 (54.4)	
Unknown	44 (17.3)	7 (17.9)	6 (21.4)	16 (13.3)	15 (22.1)

**Table 2:** The Association of Fatty Infiltration of Pancreas With the Presence or Absence of Presumed Risk Factors (N = 265)

Demographic	n (%)	PS100 ± SD	*P*
Sex			
Male	67 (26.3)	92.26 ± 16.45	.459
Female	188 (73.7)	90.43 ± 19.9	
Age			
<70 y	158 (62.7)	93.99 ± 15.58	.01
≥70 y	94 (37.3)	88.13 ± 19.84	
Body mass index			
<25	64 (29.8)	96.83 ± 9.40	.015
≥25	151 (70.2)	90.79 ± 18.69	
Diabetes mellitus			
No	156 (73.2)	92.78 ± 15.70	.629
Yes	57 (26.8)	91.53 ± 19.44	
Hypertension			
No	65 (30.5)	93.07 ± 15.28	.411
Yes	148 (69.5)	91.82 ± 17.36	
Hyperlipidemia			
No	115 (70.6)	94.61 ± 14.01	.044
Yes	48 (29.4)	88.97 ± 22.40	
Hypertriglyceridemia			
No	126 (77.3)	93.29 ± 16.64	.676
Yes	37 (22.7)	92.02 ± 14.82	
Fatty liver			
No	205 (81.0)	92.51 ± 16.64	.379
Yes	48 (19.0)	90.12 ± 18.04

**Table 3:** The Relationship of Fatty Infiltration of Pancreas With MetL (5 Components of Metabolic Syndrome + Fatty Liver) Components Separated by Sex and Age Above/Below 70

Male				Female			
Demographic	n (%)	PS100 ± SD	*P*		n (%)	PS100 ± SD	*P*
Metabolic syndrome				Metabolic syndrome			
0	5 (26.3)	88.04 ± 42.14	.628	0	17 (29.8)	98.59 ± 6.70	.06
≥3	14 (73.7)	93.62 ± 17.23		≥3	40 (70.2)	90.16 ± 18.13	
Body mass index				Body mass index			
<25	11 (20.0)	97.96 ± 10.94	.271	<25	52 (32.5)	96.80 ± 9.12	.023
≥25	44 (80.0)	91.22 ± 19.26		≥25	108 (67.5)	90.57 ± 18.46	
Diabetes mellitus				Diabetes mellitus			
No	40 (72.7)	93.06 ± 14.14	.524	No	116 (73.4)	92.69 ± 16.26	.877
Yes	15 (27.3)	89.55 ± 26.0		Yes	42 (26.6)	92.23 ± 16.84	
Hypertension				Hypertension			
No	40 (72.7)	91.48 ± 19.01	.876	No	50 (31.6)	94.59 ± 14.12	.291
Yes	15 (27.3)	92.34 ± 17.78		Yes	108 (68.4)	91.63 ± 17.28	
Hyperlipidemia				Hyperlipidemia			
No	29 (72.5)	93.40 ± 17.18	.74	No	86 (69.9)	95.01 ± 12.85	.013
Yes	11 (27.5)	95.36 ± 14.64		Yes	37 (30.1)	87.07 ± 21.62	
Hypertriglyceridemia				Hypertriglyceridemia			
No	30 (73.1)	93.07 ± 17.49	.78	No	96 (78.7)	93.36 ± 16.47	.491
Yes	11 (26.8)	94.72 ± 13.61		Yes	26 (21.3)	90.88 ± 15.41	
Fatty liver				Fatty liver			
No	51 (76.1)	88.35 ± 21.79	.128	No	154 (82.8)	93.89 ± 14.37	.017
Yes	16 (23.9)	97.04 ± 9.92		Yes	32 (17.2)	86.66 ± 20.21	
MetL				MetL			
<3	37 (67.3)	91.37 ± 19.2	.487	<3	106 (66.3)	95.01 ± 13.04	.008
≥3	18 (32.7)	95.01 ± 15.54		≥3	54 (33.7)	87.85 ± 20.54	

Male age < 70 y				Female age < 70 y			
	n (%)	PS100 ± SD	*P* (age adjusted)		n (%)	PS100 ± SD	*P* (age adjusted)
MetL				MetL			
<3	19 (59.4)	94.38 ± 11.05	.662	<3	67 (62.6)	98.02 ± 9.84	.002
≥3	13 (40.6)	96.45 ± 15.49		≥3	40 (37.4)	88.41 ± 20.94	

Male age ≥ 70 y				Female age ≥ 70 y			
	n (%)	PS100 ± SD	*P* (age adjusted)		n (%)	PS100 ± SD	*P* (age adjusted)
MetL				MetL			
<3	18 (78.3)	88.20 ± 25.12	.8	<3	39 (73.6)	89.85 ± 16.07	.5
≥3	5 (21.7)	91.29 ± 16.83		≥3	14 (26.4)	86.22 ± 20.05	

Plotting the PS100 data according to age and sex revealed an overall increase in FIP (Spearman correlation = −0.2, *P* = .001). Similar to men of all ages, PS100 decreased steadily with age in women younger than 70 years but increased with age in women older than 70 years (Figure). We then looked at the pattern of FIP in association with MetL according to the sex and age (above and below 70); in women younger than 70 years, FIP was associated with MetL (*P* = .002), but this was not the case in women older than 70 years (Table 3). In men of all ages, age remained the only predictor of pancreatic fat.

**Figure:**
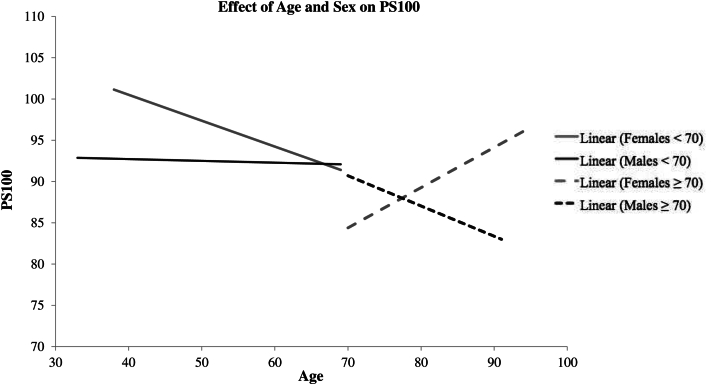
The effect of age and sex on fatty infiltration of pancreas, where PS100 is the relative fatty infiltration of the pancreas corrected to the spleen plus 100. Therefore, a lower PS100 corresponds to greater fatty infiltration of the pancreas.

## Discussion

Pancreatic cancer is a disease of ageing, and accumulating evidence suggests that FIP is a predisposing factor to this cancer.^4^^,^^6^ In this cross-sectional study of adults in the U.S., we found that age was the strongest predictor of FIP overall. We also found an association of metabolic syndrome and fatty liver with higher pancreatic fat, which was age- and sex-dependent.

Pathological and imaging studies have suggested that FIP may increase with age.^16^^,^^18^^,^^25^^,^^26^ A pathological analysis found that patients in their 30s had 10% fatty tissue in their pancreas, while those in their 80s had 35% fatty tissue in their pancreas.^27^ Another study that used ultrasound to examine the pancreas of 131 individuals showed increased pancreatic echogenicity beginning in the fourth decade of life, with most patients older than 50 years and all older than 80 years in the study having a marked increase in echogenicity and fat deposition in their pancreas.^25^ However, results from other studies provide conflicting evidence. A meta-regression of 11 studies provided evidence that FIP could be independent of age and sex.^15^ However, majority of these studies were conducted in East Asian countries, whose different environmental exposures (such as diet) and metabolic phenotype have been shown to lead to dissimilarity in the patterns of fatty liver compared with those populations from Western countries and may also contribute to the potential dissimilarities in their predictors of FIP.^28^ In a large recent study from Asia,^29^ a higher incidence of FIP was reported with age in men than in women, suggesting FIP may have a sex and age predilection. This sex difference is supported by 2 additional studies.^16^^,^^30^ Our study is the first in the U.S. to examine FIP in adults and contributes to the scarce data of pancreatic steatosis, its associated risk factors, and differences between sexes in cancer-free subjects. We used an age cutoff of 70 years because those who undergo CT colonography for colorectal cancer screening tend to be older, and the average age of our study population was 66 years. Using the age 65 years did not affect our main findings. We found pancreatic fat and its relation to age could be sex-dependent. In men, increasing age significantly correlated with greater FIP. This pattern was also seen in women younger than 70 years but not in older women, thus supporting a differential age effect on FIP according to sex.

Another suggested risk factor for pancreatic fat could be metabolic syndrome.^16^ A systematic review of 13 studies involving 49,329 subjects showed a significant association between FIP and metabolic syndrome.^31^ Mechanistically, several lines of evidence suggest that features of metabolic syndrome are associated with changes in the inflammatory milieu in the pancreas including fatty infiltration,^32^ which can lead to fibrosis and ultimately cancer. We found a sex-dependent association between pancreas fat and MetL.

Increasing number of MetL components significantly correlated with increased FIP in women younger than 70 years and not in older women or in men of any age. This novel finding suggests that risk factors for FIP could be age- and sex-dependent. This may partially explain the seemingly conflicting results in the literature regarding the effect of age and sex on pancreas fat.^15^^,^^25^^,^^27^^,^^29^ Also, our results suggest that in some subgroups (eg, older women), there may be other factors that contribute to FIP that are yet to be uncovered.

Mechanistically, ageing is shown to be associated with accumulation of senescent cells in adipose tissue in visceral organs. This can lead to further fatty accumulation in the tissue and predisposition to carcinogenesis^33^ as shown in age-associated FIP and susceptibility to PDAC development.^7^ The fatty infiltration can in turn accelerate age-related senescence, leading to a detrimental feedback loop. However, the potential role of cellular senescence in the interplay of ageing and FIP remains unknown. There is currently no evidence to suggest that the accumulation of senescent cells in the pancreas or their function in the pancreas with age could be sex-dependent.

Our study was limited because pathological correlation of fat infiltration was not possible. Instead, we relied on CT colonography. Measuring FIP on CT does not differentiate between intralobular and extralobular fat infiltration compared with biopsy. However, biopsy carries its own set of complications and may not be representative of the total fat content of the pancreas. Noncontrast CT is a noninvasive readily available tool, which is already established for colon cancer screening in real-world practice. This technique is already shown to accurately estimate organ fatty infiltration based on the objective attenuation numbers.^21^ Additionally, we can speculate that the cohort of individuals older than 70 years that are sent for CT colonography may have a better health condition than average elderly populations who would not be considered for colorectal cancer screening. Furthermore, our study is limited by the availability demographic and medical data in our system. The trend of pancreatic fat infiltration in women younger than 70 years compared with those older than 70 years is surprising, but we were unable to further investigate potential explanations because of limited records. Future prospective studies investigating the role of age, sex, and metabolic syndrome on predisposition to FIP and their possible interactions with relevant environmental exposures (such as diet and alcohol) could advance our understanding of the risk factors and mechanisms underlying FIP and associated pathologies, such as cancer.
